# The Facilitators and Barriers to Nurses’ Participation in Continuing Education Programs: A Mixed Method Explanatory Sequential Study

**DOI:** 10.5539/gjhs.v7n3p184

**Published:** 2014-11-30

**Authors:** Zohreh Shahhosseini, Zeinab Hamzehgardeshi

**Affiliations:** 1 Department of Reproductive Health and Midwifery, Nasibeh Nursing and Midwifery faculty, Mazandaran University of Medical Sciences, Sari, Iran; 2 Traditional and Complementary Medicine Research Centre, Mazandaran University of Medical Sciences, Sari, Iran

**Keywords:** Continuing Education, Mixed methods, Facilitators, barriers

## Abstract

**Background:**

Since several factors affect nurses’ participation in Continuing Education, and that nurses’ Continuing Education affects patients’ and community health status, it is essential to know facilitators and barriers of participation in Continuing Education programs and plan accordingly. This mixed approach study aimed to investigate the facilitators and barriers of nurses’ participation, to explore nurses’ perception of the most common facilitators and barriers.

**Methods:**

An explanatory sequential mixed methods design with follow up explanations variant were used, and it involved collecting quantitative data (361 nurses) first and then explaining the quantitative results with in-depth interviews during a qualitative study.

**Results:**

The results showed that the mean score of facilitators to nurses’ participation in Continuing Education was significantly higher than the mean score of barriers (61.99±10.85 versus 51.17±12.83; p<0.001, t=12.23). The highest mean score of facilitators of nurses’ participation in Continuing Education was related to “Update my knowledge”. By reviewing the handwritings in qualitative phase, two main levels of updating information and professional skills were extracted as the most common facilitators and lack of support as the most common barrier to nurses’ participation in continuing education program.

**Conclusion:**

According to important role Continuing Education on professional skills, nurse managers should facilitate the nurse’ participation in the Continues Education.

## 1. Introduction

Education as the foundation for all our learning is the most important factor to optimize human resources in that UNESCO emphasized continuing education in healthcare issues on the verge of the twenty-first century (Emamzadeh Ghasemi, 2000). Continuing education is a process that prepares the staff members for improvement and better efficacy in current or future positions, modifies their thinking and action, and furnishes them with professional information they need to achieve organizational goals (Ebadi, Vanaki, Nehrir, & Hekmatpou, 2007).

Continuing Education (CE) for medical community is one of the modern strategies to maintain and elevate knowledge in medical community, which in turn elevates the health status of the society (Shahidi, Changiz, Salmanzadeh, & Yousefy, 2010). Today, the subject of continuing professional improvement in medical sciences is followed and attended to like other professions (Ebrahimi et al., 2012). On the basis of Edinburgh Statement, the prime objective of CE is to maintain and elevate clinical functioning (Ebrahimi et al., 2012; Metcalfe, 1989). Furthermore, CE is one of the principles in subject fields related to medical sciences including nursing. Nurses need to attend CE in order to increase their skills and attain their professional goals. Studies show that knowledge gained through basic professional education has a half life of 2.5 years, and needs to be updated at the end of this period (Chong, Sellick, Francis, & Abdullah, 2011; Happell, 2004). Moreover, such training will be expired 5 years after graduation, so lack of CE can lead to poor services to patients or even patients’ death. Therefore, it is vital to update knowledge and skills of nurses through CE (Chong et al., 2011).

Nurses attend CE for several personal, professional and organizational reasons (Ebrahimi et al., 2012; Flores Pena & Alonso Castillo, 2006). These reasons include professional knowledge, professional success, change of routines, improvement in power and decision making, improvement in social welfare, improvement in social relations and gaining professional credit (Chong et al., 2011; Kristjanson & Scanlan, 1989; Waddell, 1993). Among other factors, we can mention population factors, beliefs, attitudes, motivating factors, and educational opportunities (Chong et al., 2011). The most conspicuous of these factors are improvement in professional knowledge and skills, maintaining specialty and professional methods, and improvement in personal abilities in serving people.

CE is one of the most important responsibilities of managers and has its own advantages and problems. CE can help improve professional capabilities of people; nonetheless, it can aggravate the conflicts between staff members and managers due to staff member’s lack of motivation to attend these courses and lack of organizational conditions to apply the trained subjects in the work environment (Ebrahimi et al., 2012). The literature review shows huge expenses, time consumption, unawareness of the time, and lack of managers’ support are barriers to CE. Furthermore, heavy duties in wards, lack of personnel, and poor evaluation system for nurses’ work are among the reasons why nurses allocate little time to CE. A study in Iran showed that 80% of nurses found the information taught in CE classes irrelevant to the wards they worked in, and that 60% were against this type of education (Ebadi et al., 2007; Ebrahimi et al., 2012). On the contrary, supervisor’s support availability of CE and peer encouragement are effective factors in attending CE (Glass Jr & Todd-Atkinson, 1999). Studies show that managers play an important role in helping nurses participate in CE programs in that manager’s support like rewarding the staff with or without leave of absence is a motivator for attending CE (Kersaitis, 1997). Furthermore, other motivating factors are improvement in professional skills, providing professional services, professional performance proving, interaction, personal interests, job security, professional commitment, individual’s interest to grow and develop, personal need to update information, and increase knowledge and skills (DeSilets, 1995; Ebrahimi et al., 2012; Kristjanson & Scanlan, 1989). In addition, studies show that compulsory CE has less effect in increasing nurses’ motivation to attend CE than optional CE (O’Connor, 1979). So, it is necessary to survey CE in order to facilitate and improve nursing (Chong et al., 2011; Levett-Jones, 2005).

Since several factors affect nurses’ participation in CE, and that nurses’ CE affects patients’ and community health status, it is essential to know facilitators and barriers of participation in CE programs and plan accordingly. Furthermore, it seems facilitators and barriers mutually affect one another, so it is best to study them together. Conducting qualitative study in combination with quantitative study can help attain a better understanding of the social context and individual’s perception of facilitators and barriers of nurses’ participation in CE (Creswell & Plano Clark, 2011). Therefore, this mixed approach study aimed to investigate the facilitators and barriers of nurses’ participation, to explore nurses’ perception of the most common facilitators and barriers to participate in CE.

## 2. Methods

### 2.1 Ethical Approval

All participants were informed about the purposes and the methods of the research. They were informed that participation in the study is voluntary and that they can refuse to participate or withdraw from the study without any penalties. Moreover, the participants were reassured that their responses were kept confidential and their identities were not be revealed in research reports or in the publication of the findings. The Ethics Committee of Mazandarn University of Medical Sciences had approved this protocol.

**Figure 1:**
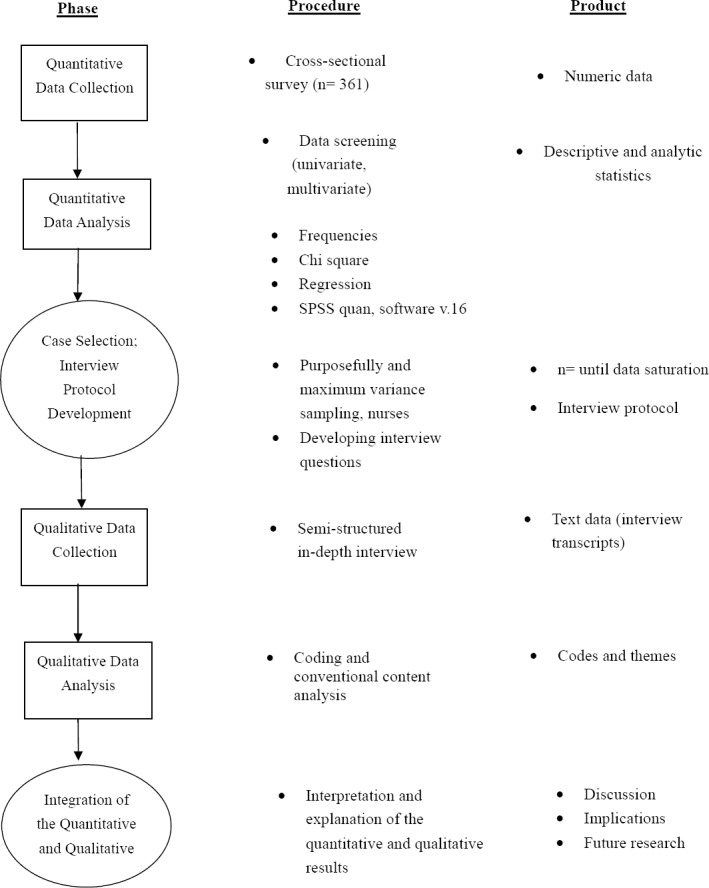
Study visual diagram

The current study was a research with sequential explanatory mixed methods with follow up variant design. The Figure 1 showed study visual diagram. This study had two phases:

### 2.2 First Phase: Quantitative Phase

This phase was a cross-sectional survey. The objective of this phase was to determine the facilitators and barriers of nurses’ participation. This study was conducted among 361 nurses in Mazandaran University of Medical Sciences, Iran in 2013. Convenience sampling was used as the sampling method.

#### 2.2.1 Sample size and Sampling Methods

The estimated sample size of this study was 355 nurses in Mazandaran University of Medical Sciences, Iran.

To determine the sample size in the quantitative phase, the following calculation was done:


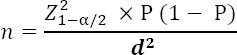


With regard to type 1 error (alpha) of 5% and accuracy of 0.5 and the most common influencing factors of nurses’ participation in continuing education programs in Tabriz University of Medical Sciences (tendencies to learn and increase their professional knowledge) of previous researches that has been 64.2% (Ebrahimi et al., 2012), approximately 355 samples were calculated. In this study 361 participants completed questionnaire.

#### 2.2.2 Instruments

The research instrument was a questionnaire conducted by researchers. This questionnaire was two parts. First part was related to socio demographic information (11 questions). Second part was questions about facilitators’ factors (16 questions) and barriers’ factors (20 questions). The second part had questions with five score of lickers’ scale.

The questionnaire validity was determined with face and content validity. The target group opinion (Impact Score) was used to explore face validity. Content Validity Ratio (CVR) and Content Validity Index (CVI) were used to determine content validity. The convenience sample of 20 nurses complete questionnaire twice in a fortnight to evaluate the reliability of the questionnaire. In order to evaluate the questionnaire’s reliability, the Intraclass Correlation Confficient (ICC) is calculated for all domains and items. Internal consistency of the questionnaire in different dimensions was evaluated using Cronbach’s alpha coefficient.

None of the items were considered unacceptable by using impact score (upper than 1.5), CVI (0.90) and CVR (0.61). Based on the results of the pilot study, and 10 experts’ comments, minor changes were made and questionnaire was found appropriate for the Iranian society.

At next stage, test-retest and internal consistency were used to evaluate its reliability. To this end, 20 nurses were selected. They completed questionnaire anonymously and after two weeks, the same group completed it again. Test re test was used to evaluate the questionnaire’s reliability. The Intraclass Correlation Coefficient (ICC) was calculated for all domains and items in this study. In this study the mean of ICC was calculated 0.93. Qualitative indexes for ICC were determined as follows: <0.4 (weak reliability), 0.4-0.6 (average reliability), 0.6-0.8 (good reliability), and 0.8-1 (excellent reliability) (Corson, Boyd, Kind, Allen, & Steele, 1999). Cronbach’s alpha was used to calculate internal consistency. Cronbach’s alpha coefficient = 0.92 was reported in this study (Hamzehgardeshi & Shahhosseini, 2014).

#### 2.2.3 Statistical Analysis

Collected data were analyzed using Statistical Package for Social Scientist version 16 for windows (SPSS Inc., Chicago, USA). Descriptive and analytical indicators were used to express the data. The chi-square test was used for the univariable analysis, and logistic regression was used for multivariable analysis.

### 2.3 Second Phase: Qualitative Phase

This phase of the study was done with the aim of exploring nurses’ perception of the most common facilitators and barriers to participate in CE. This phase was done using the conventional content analysis (CA) approach.

#### 2.3.1 Sampling Method

Purposeful sampling followed by maximum variance sampling was conducted among the nurses. Purposeful sampling guided the researcher to find nurses with pure experiences regarding the most common facilitators and barriers to participate in CE. In this regard, introductory pilot interviews was employed in order to find the nurses who provide a breadth and depth response. The selected nurses participated in in-depth interviews.

#### 2.3.2 Data Collection

Semi-structured in depth interviews were the researchers’ instrument to obtain information about the participants’ experiences regarding the most common facilitators and barriers to participate in CE. Firstly, the researcher provided the list of interview questions, and tried to improve his interview techniques using his/her research team advice. Interview questions include “Please describe your feeling while doing this facilitator and barrier”, “What causes these facilitator and barrier?”, “Why and how? Explain more”. Gathered data were analyzed in order to find themes and categories from the in-depth interviews.

#### 2.3.3 Research Design

Qualitative Content Analysis was used in our project. Content Analysis was a qualitative research method because it was exploratory by nature. Qualitative and quantitative content analyses are two types of content analysis. Qualitative content analysis emphasises interpretation, subjectivity, flexibility in process, and concern for influence of context on the research process (Creswell & Plano Clark, 2011; Given, 2008; Holdford, 2008).

#### 2.3.4 Data Analysis

The research team reviewed the in-depth interviews, and extracted codes and categories to assess the accuracy of the coding process. The qualitative and conventional content analysis approach was used, in which themes and categories were explored to reveal the nurses’ experiences of most common facilitators and barriers to participate in CME. Moreover, the constant comparison method was used during the research. In this study, the conventional qualitative content analysis was used in which the categories are derived from the data and not from a primary theory (Holdford, 2008). A computer-assisted program (MAXQDA 10) was used for data management (Kuckartz, Kuckartz, & Andaluces, 2002).

## 3. Results

### 3.1 First Phase: Quantitative Phase

Of the 361 participants, 93.94 percent of them were female, and the rest were male. The mean and standard deviation of participants’ age and their employment record were 37.14±7.58 and 11.48±4.47 years respectively.

Based on the findings of this study, the mean score of facilitators to nurses’ participation in CE was significantly higher than the mean score of barriers (61.99±10.85 versus 51.17±12.83; p<0.001, t=12.23). The results of this research showed that the highest mean score of facilitators of nurses’ participation in CE was related to “Update my knowledge” (Table 1). Also the highest mean score of barriers according to participants’ point of view, in three personal, interpersonal and structural domains, were related to “Time constraints”, “Lack of co-workers’ support” and “work commitments”, accordingly (Table 2).

**Table 1:** Mean score and standard deviation of facilitators and barriers to nurses’ participation in ce

Facilitators		Mean	Standard Deviation
**Personal barriers**	Time constraints	3.24	1.18
	Domestic responsibilities	2.86	1.18
	Emotional stress	2.12	1.16
	Poor physical health	1.91	1.10
**Interpersonal barriers**	Lack of co-workers’ support	2.74	1.17
	Negative experiences with previous CE programs	2.29	1.15
	Lack of family support	2.23	1.20
	Poor interaction of CE programs’ staff	1.91	1.12
**Structural barriers**	Work commitments	3.50	1.24
	Cost of courses	3.32	1.17
	Geographic distance	3.03	1.25
	Poor scheduling of CE programs	3.00	1.15
	Lack of organizational support	2.95	1.29
	Lack of information about provided CE programs	2.93	1.18
	Lack of accessibility to provided CE programs	2.91	1.12
	Lack of supervisors’ support	2.89	1.23
	Lack of relevant CE programs	2.82	1.07
	Poor quality of provided CE programs	2.49	0.99
	Needs satisfied by on the job training	1.95	1.09

**Table 2:** Analysis of variance between barriers to nurses’ participation in ce

Barriers	N	Mean± SD	95% Confidence Interval for Mean	F	Sig
**Personal**	361	10.15±3.36	9.80-10.50	2122.66	p<0.001
**Interpersonal**	361	9.18±3.37	8.83-9.53		
**Structural**	361	31.83±7.80	31.02-32.64		
**Total**	1081	17.05±11.71	17.75-17.75		

In order to answer this question that what is the most priority domain of barriers to participation in CE among Iranian nurses? A one-way analysis of variance was conducted. The results showed the mean score of personal and structural barriers was significantly higher than the mean score of interpersonal ones (F=2122.66, p<0.001).

#### 3.2 Second Phase: Qualitative Phase

Totally, 25 nurses including 15 females and 10 males, among whom two people had a master’s degree and 23 had a bachelor’s degree, participated. Two main categories were extracted from the interviews: updating information and professional skills as the most common facilitator and insufficient support as the most common barrier to nurses’ participation in continuing education programs. In addition, 6 subcategories of updating information, increasing clinical skills, lack of colleague’s support, work overload due to professional commitments and insufficient fund were also classified.

**Theme 1: Updating knowledge and professional skills**

This main level was extracted from two categories such as updating knowledge and increasing clinical skills.

**Updating knowledge**

From the participants’ view, high motivation for updating nursing knowledge was expressed as the most important and most common facilitator of nurses’ participation in continuing education program. In fact, some of the participants also expressed mandatory continuing education for nurses. From participant’s perspective, they are required to participate in special courses of continuing education for their annual assessment and promotion. In this regard, participant 12 who is a 35-year-old male purports that:

“We need to pass the required hours of continuing education program for our annual promotion. Of course, most of the time, it is not due to the obligatory nature of the courses but we participate continuing education programs in order to update our information and professional knowledge even when the classes are held in other provinces.”

**Increasing clinical skill**

Most of the participants considered the increase in clinical skills as their most common motivation to participate in continuing education program. In this regard, participant 22 who is a 27-year-old woman expressed:

“In my opinion, all the continuing education programs are not interesting. Some of them just take our time. But there are programs that have an applicable design and implementation. These programs enhance our practical skills. When such programs are held at university, I am so motivated to participate in them. This is because in this sense, I can leave my routine state and learn something new which could help me in my clinical practices.”

**Theme 2: Inadequate support**

This main category was extracted from 3 subcategories: lack of colleague’s support, work overload due to professional commitments and insufficient fund.

**Lack of colleague’s support**

Some of the participants, specifically the nurses who had less than 5 years of experience expressed the lack of their colleagues’ support as their most important barrier in participating in continuing education program. In this sense, participant 25, a 25-year-old woman nurse said: “To my mind, the support of more experienced colleagues is important for us as younger nurses. I mean we younger nurses cannot comment much on our monthly plan, for instance we cannot ask for a leave on conference days. That is why we have troubles in switching shifts, since most of the time the colleagues who have higher experiences aren’t flexible in switching their shifts, which prevents us from participating in programs.”

**Work overload due to professional commitments:**

Most participants stated the high work overload as one of the most important barriers to participating continuing education program. Data reveals that some nurses are in charge of several responsibilities. Therefore, professional commitments and high workload prevents their participation in continuing education program. Participant 9, a 40-year-old woman nurse said: “I, in addition to being the head nurse of the psychology ward, am a member of several committees in the hospital. Moreover, in most cases that the hospital faces limits of working supervisors, I have to cooperate and be the supervisor for a couple of shifts. So, usually, I don’t find time to participate in continuing education program.”

**Insufficient fund**

Most participants expressed the lack of holding proper educational programs due to insufficient fund as one of the main barriers to lack of participation in continuing education program. This was in a way that they considered providing the program budget as one of the most important problems of program executors. In addition, nurses had objections on paying the registering fees on their own. In this regard, participant 10, an educational supervisor who is a 38-year-old woman stated:

“Expenses of implementing a proper and applicable educational program are very high. These expenses include the instructors’ tuition fees, catering and providing equipment and educational facilities. How many times a year do you think it is possible to hold nurses’ continuing education program by having relationships and instructors’ cooperation by not receiving tuition fees? Moreover, if nurses are supposed to pay for registration, still it isn’t possible as nurses would object to this issue and would say why they need to pay the registration fee for obligatory continuing education program while the hospital itself should provide all the expenses.”

## 4. Discussion

The quantitative results showed that the mean score of facilitators to nurses’ participation in CE was significantly higher than the mean score of barriers. The results of this research showed that the highest mean score of facilitators of nurses’ participation in CE was related to “Update my knowledge”. Also the highest mean score of barriers according to participants’ point of view, in three personal, interpersonal and structural domains, were related to “Time constraints”, “ Lack of co-workers’ support “ and “ work commitments “.

By reviewing the handwritings in qualitative phase, two main levels of updating information and professional skills were extracted as the most common facilitators and lack of support as the most common barrier to nurses’ participation in continuing education program. In addition, 6 subcategories of updating knowledge and increasing clinical skills, time limit, lack of colleagues’ cooperation, workload due to professional commitments and insufficient fund were extracted.

Being consistent with other studies (Ebrahimi et al., 2012), the results of quantitative study revealed that updating knowledge was the most common facilitator of nurses’ participation in continuing education programs. In line with results of quantitative study, the findings of qualitative study also showed that from the nurses’ perspective, updating knowledge and increasing clinical skills are the most important facilitator of their participation in continuing education programs. Based on previous studies, participating in continuing education programs to update one’s information was stated as one of the most effective factors of nurses’ participation in continuing education programs (Ebrahimi et al., 2012; Hossini, Moghimib, Karimic, Momenid, & Sadat, 2012). Moreover, in Nalle et al. study, the increase in knowledge and skill is of the benefits of participation in continuing education programs from the perspective of 83% of the nurses (Nalle, Wyatt, & Myers, 2010).

It should be mentioned that results of the present study is inconsistent with Vahidshahi et al. study. This is because in the mentioned study, nurses stated 60% of their primary motivation to participate in reeducation programs to earn points and 28% of them to consolidate their prior information and gaining new information (Vahidshahi, Mahmoudi, Shahbaznezhad, & Ghafari Saravi, 2007). This inconsistency could be due to the differences in the implemented programs because in recent years, by proper planning, the quality of continuing education programs has improved. Therefore, although there is a need to participate in continuing education programs to gain job promotion but many nurses wish to improve their knowledge and professional skills (Hossini et al., 2012).

The findings of the quantitative study, in line with other studies, showed that insufficient fund and expense of participation in the program were the barriers to the nurses’ participation in continuing education programs (Ebrahimi et al., 2012). In Flores Peña’s study, as well, there is a significant relationship between the monthly income and hours of participation in continuing education programs (Ebrahimi et al., 2012; Flores Peña & Alonso Castillo, 2006). Moreover, in related studies, inadequate support of the organization for achieving continuing education for nurses was shown (Gallagher, 2007; Hegney, Tuckett, Parker, & Robert, 2010; Vaezi & Vanaki, 2012).

At one end, the results of the quantitative study showed that among the structural factors, time constraint and lack of co-workers’ support and work commitments were the most important barriers of nurses’ participation in continuing education program. On the other end, findings of the qualitative study showed that from nurses’ perspective, lack of colleagues’ support and workload due to work commitment were the most important barriers to their participation in continuing education programs. A review of the previous studies showed that from the nurses’ perspective, setting nurses’ working shifts, lack of the possibility to request leave times from workplace to attend the program were great barriers for nurses to participate in continuing education programs (Ebrahimi et al., 2012; Flores Peña & Alonso Castillo, 2006; Schweitzer & Krassa, 2010). In similar studies, nurses’ wok pressure and creating work pressure for colleagues during participation in continuing education programs are mentioned as the barrier to participating in continuing education (Penz et al., 2007; Vaezi & Vanaki, 2012).

In line with other studies, the results of the present study showed that updating knowledge and increasing clinical and professional skills are the most important facilitators for nurses’ participation in continuing education programs (Ebrahimi et al., 2012). According to Nalle et al. study, personal and professional interests were the most important reasons for participation in continuing education programs (Nalle et al., 2010). Therefore, nurses wish to attend programs that are useful for their present and future career (Flores Peña & Alonso Castillo, 2006).

Continues Education for nurses and other medical community is an important issue for their scientific and professional development (Ebrahimi et al., 2012; Shahidi et al., 2010). The study findings similar to previous studies showed two important limitations to the successful Continues Education include inhibitors of formal education programs and lack of changes according to programs (Nolan, Owens, & Nolan, 1995). Several factors inhibited the nurse staff participation in Continues Education include lack of awareness, lack of manager encouragement, money, and family barriers (Kristjanson & Scanlan, 1989; Nolan et al., 1995).

## 5. Conclusion

Based on the results of this study, the basis for nurses’ participation in programs for their job promotion should be provided. Therefore, educational programs should be developed and implemented based on nurses’ professional needs. In addition, due to the barriers to nurses’ participation in continuing education programs such as lack of colleagues’ support, fund limits and nurses’ work commitments which are barriers for their presence, E-learning and distance education should be developed more. This would result in improvements on the quality of patients’ care which is the main pillar in nursing profession. According to important role CE on professional skills, nurse managers should facilitate the nurse’ participation in the Continues Education.
